# Mast Cells and Basophils in Major Viral Diseases: What Are the Correlations with SARS-CoV-2, Influenza A Viruses, HIV, and Dengue?

**DOI:** 10.3390/cells13242044

**Published:** 2024-12-11

**Authors:** Luca Gammeri, Serena Sanfilippo, Clara Alessandrello, Sebastiano Gangemi, Paola Lucia Minciullo

**Affiliations:** Department of Clinical and Experimental Medicine, School and Operative Unit of Allergy and Clinical Immunology, University of Messina, 98125 Messina, Italy; luca.gamm1993@gmail.com (L.G.); serenasanfilippo.doc@gmail.com (S.S.); clara.alessandrello@outlook.it (C.A.); sebastiano.gangemi@unime.it (S.G.)

**Keywords:** adaptive immune response, innate immune response, mast cells, basophils, COVID-19, HIV, dengue, influenza virus

## Abstract

The SARS-CoV-2 pandemic has significantly impacted global health and has led the population and the scientific community to live in fear of a future pandemic. Based on viral infectious diseases, innate immunity cells such as mast cells and basophils play a fundamental role in the pathogenesis of viral diseases. Understanding these mechanisms could be essential to better study practical therapeutic approaches not only to COVID-19 but also to other viral infections widely spread worldwide, such as influenza A, HIV, and dengue. In this literature review, we want to study these concepts. Mast cells and basophils intervene as a bridge between innate and acquired immunity and seem to have a role in the damage mechanisms during infection and in the stimulation of humoral and cellular immunity. In some cases, these cells can act as reservoirs and favor the replication and spread of the virus in the body. Understanding these mechanisms can be useful not only in therapeutic but also in diagnostic and prognostic perspectives. The prospects of applying artificial intelligence and machine learning algorithms for the creation of very accurate diagnostic/prognostic tools are interesting.

## 1. Introduction

The COVID-19 pandemic has had a huge impact worldwide, especially regarding the mortality rate. Great interest is involved in understanding the pathogenetic mechanisms behind this condition to try to find prognostic parameters of disease that can predict severity.

In a previous work, we assessed the possibility that serum and tissue variations in basophils and mast cells could be predictors of disease severity. In detail, we observed that a reduction in basophils in the acute phase of the disease was associated with more severe COVID-19. At the same time, mast cells could represent a therapeutic target implicated in airway inflammation [1]. Furthermore, mast cells in the nasal and lung epithelium were associated with cytokine storms, inducing pulmonary fibrosis and thrombosis [2]. 

In recent years, the interest in pandemic or epidemic infectious diseases has gradually increased, especially in light of the growing fear of future pandemics.

In light of the potential role of these cells in the pathogenesis of SARS-CoV-2 disease, their involvement in other widely spread viral infectious diseases could be of great interest. In this work, we focused our attention on COVID-19 and other pandemic infections and the role of mast cells and basophils in the pathogenic process. In particular, research has focused on the human immunodeficiency virus (HIV), influenza A viruses (IAVs), and dengue virus, given the recent increase in cases. Furthermore, we wanted to provide an update on the role of these two cell populations in SARS-CoV-2 infection.

Our review aims to better understand the role of mast cells and basophils in the physiopathological mechanisms of the main viral diseases responsible for pandemics or which could cause them in the future. Furthermore, we want to analyse the potential role of these cells as prognostic indicators and possible therapeutic targets.

## 2. Materials and Methods

We performed a strategic search through PubMed to find studies regarding the role of basophils and mast cells in SARS-CoV-2, HIV, IAV, and dengue virus infection. The keywords used for the search were “basophils/mastocytes” and “HIV” or “SARS-CoV-2” or “COVID-19” or “Influenza A” or “Dengue”. We found 1250 manuscripts. We selected articles in English published up to October 2024. Considering the studies on COVID-19, we have only selected articles published after October 2021, as the previous ones were the subject of a previous review by us. The same criterion was adopted considering the topic “HIV”, since a review from 2016 is present in the literature. Therefore, we limited the search to all studies published after December 2016. After excluding duplicates, we analysed the abstract and text for each remaining article and selected 58 valuable articles. Of these studies, thirty-six involved mast cells, sixteen involved basophils, and six involved both cell types.

## 3. Results

### 3.1. Role of Mast Cells and Basophils in the Course of SARS-CoV-2 Infection

Mast cells are important sentinel cells of our immune system. They are strategically located at sites that interface with the external environment, such as the skin and mucosal surfaces of the respiratory tract and nasal cavity, to protect the body against viral and bacterial entry. 

Although mast cells are known as effectors of the allergic response, they can interact with various immune cells by releasing soluble factors or by direct contact to regulate the immune response in a beneficial or harmful way. Several pathogens or their products can activate mast cells, inducing the secretion of cytokines and chemokines by degranulation-dependent or degranulation-independent pathways. The release of these inflammatory mediators contributes to the recruitment of immune effector cells to remove pathogens; however, these mediators may also promote inappropriate proinflammatory responses, contributing to the damage of epithelial–endothelial barriers and facilitating pathogens’ invasion [3].

SARS-CoV-2, through the spike protein, binds to angiotensin-converting enzyme 2 (ACE2) to enter cells, and then host serine protease transmembrane protease serine 2 (TMPRSS2) cleaves the spike protein and enables cellular membrane fusion. ACE2 and TMPRSS2 are expressed on the epithelium of the lungs, skin, and intestines. In contrast, other host molecules that may be involved in SARS-CoV-2 invasion, such as CD147 and CD26, are expressed in both epithelium and immune cells [4]. 

The host response to viral RNA invasion activates Toll-like receptor (TLR)-3 on mast cells with type I IFN production (beneficial effect); however, the virus can cause sensitization of mast cells with IgE production that bind to the FcεRI receptor, triggering an excessive proinflammatory reaction (harmful effect). Overall, the RNA virus causes the production of IFN-gamma e CXCL8, leading to the recruitment of NK cells that also contribute to type I IFN production. The virus can stimulate mucosal mast cells, causing the release of TNF-alpha, IL-1, IL-6, proteases (worsening the inflammatory condition), histamine, prostaglandin D2, and leukotriene C4 (inducing acute bronchoconstriction and lung inflammation) [5].

The main inflammatory cytokines and chemokines widely produced by patients with severe forms of COVID-19 are IL-6, IL-8, IL-1β, TNF-alpha, IFN-gamma, MIP1α and 1β, CCL2, CCL5, CCL20, CXCL1, CXCL2, CXCL8, CXCL10, and CXCL17. In particular, high levels of IL-6, TNF-alpha, and C-reactive protein (CRP) appear to be independent factors for the severity of COVID-19 disease [6,7,8,9,10].

Patients with mild symptoms after COVID-19 can show diffuse, multiorgan symptoms several months after the infection. These symptoms were initially reported in children and named Multisystem Inflammatory Syndrome (MIS-C), but a similar condition has also occurred in adults, named Multisystem Inflammatory Syndrome (MIS-A). The typical symptoms (malaise, chest tightness, myalgias, brain fog, and other neuropsychiatric symptoms) are very similar to those associated with Mast Cell Activation Syndrome [11]. 

Inspired by Murdaca et al.’s earlier work in 2021, we assessed the current state of the art in the literature on the role of mast cells in COVID-19 [1].

In a study by Li et al. [12], it was observed that patients with frailty were more prone to poor health conversions and sequelae (these adverse health transitions were significantly associated with mast cell activation).

In a mouse and non-human primate model studied by Tan et al. [13], the widespread activation of mast cells in lung tissue (densely localized and degranulating, especially within the haemorrhagic regions of infected lung) was noticed. 

According to this, Gebremeskel et al. [14] have evaluated the serum of 19 SARS-CoV-2 patients and 20 uninfected controls, showing that mast cells-derived proteases and eosinophil-associated mediators are increased in COVID-19 lungs and/or serum; they also observed that mast cells activation is induced through TLR3/7/8 stimulation.

Recently, Cao et al. [15] confirmed that SARS-CoV-2 infection induces the accumulation and degranulation of mast cells in the peritrachea of mice. Indeed, mast cell activation induces the production of inflammatory factors in bronchial epithelial cells.

Otherwise, in a multicentre prospective study by MacCann et al. [16], 61 patients with COVID-19 were compared with 54 healthy controls, showing a greater downregulation of FCER1A (gene encodes for FcεRI on mast cells) in the moderate/severe COVID-19 group than the group with mild disease; a downregulation of the TNF-alpha, PTGS2, and IL1B genes in the moderate/severe COVID-19 group was also noted, suggesting the downregulation of mast cells’ activation. These events lead to a dysregulation of the antiviral response.

To verify that the interaction between spike protein and ACE2 is pivotal to activating mast cell degranulation, Wu et al. [17] attempted to block the interaction through prior treatment with an anti-ACE2 antibody, finding that spike-RBD treatment of LAD2 cells was no longer able to induce mast cells’ degranulation.

The use of mast cell stabilizers (sodium cromoglicate) and antihistamines could explain the lower incidence of severe forms of COVID-19 in atopic patients, so mast cells could potentially be a therapeutic target to avoid a worse prognosis [17]. In addition, using quercetin supplements could also modulate the inflammatory system by reducing the risk of developing severe forms of COVID-19 [18].

Murdaca et al.’s review also focused on the role of basophils in the pathogenesis of the disease, analysing the studies present in the literature up to 2021 [1]. The number of basophils is depleted during acute infection and the efficacy of humoral and cellular immune responses to SARS-CoV-2 is consequently compromised. In basophils, a reduction in the expression of surface CRTH2, a receptor for prostaglandin D2, and a significant increase in programmed cell death ligand 1 (PD-L1) were observed, especially in the most severe cases of disease. CRTH2 is a critical activator of the polarized T helper response.

In the chronic phase of the disease, on the contrary, basophils showed a progressive increase in levels with the improvement of the humoral immune response, perhaps related to the production of IL-4 by these cells [1].

In recent years, several authors have continued to focus on the direct correlation between basophils and the disease. Bonam et al. [19] demonstrated that SARS-CoV-2 stimulates the production of IL-13, both in resting basophils and in those primed by IL-3. In the same study, the authors found that neither infected epithelial cells nor the virus itself can alter the expression of CD69, CD13, and/or the degranulation marker CD107a, important surface markers associated with basophil activation. Furthermore, the same authors state that SARS-CoV-2 does not induce PD-L1 on basophils, contrary to what was found in past studies [19].

Recently, the coronavirus receptor (CoV-R) expression profiles have been studied in different cells of innate immunity and related cell lines. Basophils, in studies by flow cytometry, express CD13 and CD147 of the CoV-R, while they do not express CD26. Then, studying the drug effects on the expression of CoV-R, it was found that dexamethasone, vitamin D, and hydroxychloroquine do not affect the expression of these receptors [20].

In recent years, many authors have continued to observe the correlations between biohumoral profiles and the clinical characteristics of SARS-CoV-2 infection. Liu et al. [21] studied the profile of a group of patients admitted to C10 West Ward, Tongji Hospital in Wuhan City. From their retrospective analysis, it emerged that, among the various parameters considered, basophil levels were significantly elevated in critical patients. Above all, basophil levels were higher in the death subgroup compared to the survival subgroup.

By retrospectively studying the cellular profiles of patients before and after contracting the disease, it was also demonstrated that those with higher levels of basophils and monocytes were at greater risk of contracting the severe infection [22].

In contrast, in the retrospective study by Binsaleh et al. [23] on a Saudi Arabian population of 108 hospitalized COVID-19 patients, disease-related mortality was significantly associated with reduced basophils. 

In support of this, the study by Ben et al. [24] demonstrated that individuals with non-severe disease show reduced levels of basophils and basophilic aminotransferases (%) compared to healthy subjects. 

Moreover, studies by Rauf Saeed and Lal on 105 Pakistani patients did not demonstrate any correlation between basophils and disease severity [25].

In recent years, the application of artificial intelligence to studying various pathological conditions has become increasingly attractive [26]. Through machine learning (ML), we can research and combine large amounts of data and create precise predictive algorithms. Many authors have exploited these tools to obtain instruments capable of predicting the risk of hospitalization or mortality from disease.

Fu et al. [27] applied the LASSO logistic regression-based predictive model to a database of 1929 patients with COVID-19. The ML model identified 28 variables useful for the prognosis of critical illness, and among them, a low basophil count is associated with the development of critical illness at the time of hospital admission.

Chadaga et al.’s model also allowed the use of different peripheral blood markers to stratify SARS-CoV-2 patients’ risks, with overlapping results [28].

Unsupervised clustering analysis with neural network self-organizing maps was used to identify potential variables in routine blood tests that can help identify COVID-19 infection at the time of hospital admission. Variables such as the number of leukocytes, basophils, eosinophils, and red blood cell distribution width showed a strong influence on the clustering performance [29].

Wang and his group [30] developed and validated a multivariate model based on complete blood count to predict recovery from moderate disease. Their model used data from 86 patients and 38 patients from another hospital for external verification of the model. The logistic regression model identified basophil levels’ mean corpuscular volume, red blood cell distribution width, and platelet distribution width as valuable parameters for predicting recovery. Indeed, small increases in these parameters within the normal range correspond to clinical improvement in these patients.

Min Baik et al. [31] developed models based on eXtreme gradient boosting, category boosting, and light gradient boosting machines to easily differentiate COVID-19 pneumonia from other forms of pneumonia. Among the various parameters used, basophil seemed to be one of the most important features in differentiating the two groups of diseases.

**Figure 1:**
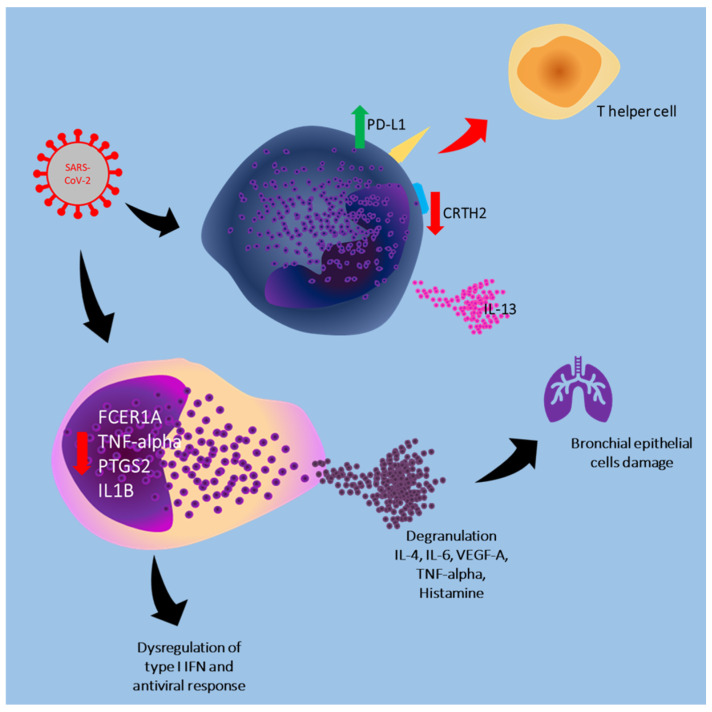
In the active phases of infection, the virus causes a reduction in the expression of CRTH2 on the surface of basophils and a significant increase in PD-L1, reducing the activation of the polarized T helper response. Active mast cells of the bronchial mucosa release TNF-alpha, IL-1, IL-6, proteases, histamine, prostaglandin D2, and leukotriene C4, promoting lung damage. In addition, the altered gene expressions induced by the virus significantly reduce the antiviral response of the immune system dependent on type I INF; The green arrow indicates an increase, the red arrows indicate a decrease.

Figure 1 summarizes the mechanisms through which SARS-CoV-2 interacts with mast cells and basophils.

Table 1 summarizes data from studies on the role of mast cells and basophiles.

### 3.2. Role of Mast Cells and Basophils in the Course of Influenza a Virus Infection

Influenza viruses cause recurrent upper and/or lower respiratory tract diseases, with symptoms ranging from simple nasopharyngitis to pneumonia. It can also cause acute respiratory distress syndrome and rarely lead to death from respiratory failure. Influenza viruses usually appear in more or less large population groups (epidemic outbreaks), but they can sometimes spread to a larger population and cause pandemics. Viruses that can cause contagious respiratory diseases are influenza A (IAV) and influenza B (IBV), belonging to the Orthomyxoviridae family [32]. These viruses are divided into subgroups based on the antigenic properties of their surface proteins: hemagglutinin and neuraminidase. IAVs are not exclusive to humans but can infect animals and subsequently be transmitted to humans through zoonoses. IAVs tend to spread periodically as seasonal viruses, causing more or less extensive epidemic outbreaks. However, in the past, IAVs have been responsible for four pandemics: the “Spanish flu” of 1918 (H1N1), the “Asian flu” of 1957 (H2N2), the “Hong Kong flu” of 1968 (H3N2), and the influenza A pdm09 from 2009 to 2010 (H1N1) [33].

The innate immune response to IAVs is complex and poorly understood. The protagonists of the immune response can be responsible for abnormal responses, which can cause serious clinical conditions.

In literature, several authors have studied the role of different cells of innate immunity in the pathogenesis of the infection, often to find valid therapeutic targets.

The role of mast cells in the innate response against influenza viruses is unclear. Still, different viruses can induce distinct antiviral gene expression and cytokine profiles in mast cells in response to infection [34].

Mast cells play a key role in the early stages of H5N1 infection, especially in the pathogenesis of lung injury. In 2012, Hu et al. [35] studied the activity of mast cells infected by the H5N1 influenza virus in vitro (P815 cell line) and in vivo (mice). The virus can activate the mast cell, stimulating its proliferation and the release of inflammatory mediators such as histamine, tryptase, and especially IFN-gamma.

IFN-gamma released in large quantities induces apoptosis of lung epithelial cells, causing lung damage.

Subsequently, Graham et al. [36] identified a unique inflammatory cascade activated during influenza A virus infection. Indeed, in this study, A/WSN/33 induced mouse mast cells release of histamine, chemokines, cytokines, and leukotrienes. This cytokine production is dependent on RIG-I/MAVS, whereas histamine production is independent of this mechanism. Furthermore, in vivo studies have shown that respiratory infection with the same virus induces a mild disease in genetically mast cell-deficient mice.

Moreover, another pathway involved is TRL-3. A study on P815 cells infected with the influenza A virus has demonstrated how this pathway determines the production of proinflammatory cytokines and chemokines such as IL-6, IFN-gamma, TNF-alpha, CCL-2, CCL-5, and IP-10 [37]. Furthermore, according to the same authors, mast cells, in addition to actively participating in the immune response, would be a reservoir for viral replication.

Liu et al. [38] tried to analyse the inflammatory response following influenza A infection in the P815 cell line. H5N1 and H7N2 viruses would induce mast cell apoptosis through the intrinsic mitochondria/cytochrome c-mediated pathway. Furthermore, the authors demonstrated how inhibiting the virus-induced apoptotic mechanism reduced proinflammatory cytokines and viral replication.

Desheva and her team [39] obtained a contrasting result. They studied the role of these cells in immunized mice compared to a control group. Vaccination significantly reduces the risk of lethal infection. Furthermore, when comparing the lungs of vaccinated mice to the control group, the number of mast cells and the amount of released histamine were significantly increased.

Demonstrating the key role of mast cells in the pathogenesis of airway damage following influenza A virus infection is a study on the efficacy of sodium cromoglicate in the treatment of the disease. Sodium cromoglicate is a mast cell stabilizer. Han et al. [40] studied the airways of mice infected with the H5N1 virus and treated with sodium cromoglicate. Not only did these mice have a higher survival rate from the disease, but reduced expression of IL-6, TNF-alpha, Toll-like receptor 3, and TRIF (TIR domain-containing adaptor-inducing interferon-β) was demonstrated in the lungs of sodium cromoglicate-treated mice.

In a more recent study, Huo et al. [41] demonstrated how melatonin influences mast cells activity. In fact, melatonin is a potent antioxidant and anti-inflammatory molecule with antiviral properties. In their study, the authors noted that melatonin-deficient mice infected with H1N1 had a significantly higher mortality rate reduced by melatonin administration. Melatonin determines the down-regulation of the HIF-1 pathway and the inhibition of proinflammatory cytokines release by mast cells, inhibiting the migration and activation of neutrophils and macrophages.

This study also indirectly provides evidence of the critical role of mast cells in determining damage during influenza A virus infection.

Recently, the genetic expression of mast cells infected by Influenza A viruses was demonstrated to be different from that of non-infected cells.

Huo et al. [42] analysed different expression genes in mouse P815 cells infected by three subtypes of influenza A virus (H1N1, H5N1, and H7N2) and studied the related functions and pathways.

The authors found substantial differences between P815 cells and not-infected mast cells, particularly in H1N1- and H7N2-infected cells.

These viruses triggered many different signalling pathways, while the pathways found in H1N1 infection were limited.

This study demonstrated the essential role of 5-hydroxytryptamine (5-HT) and cGMP/PKG signalling pathways in mast cells infected with the H1N1 and H7N2 viruses.

The analysis of the mast cell proteome gave us important information about the role of these cells in the pathogenesis of influenza A. In particular, Wu et al. [43] characterized the proteome of human mast cells infected by H1N1 and H5N1 viruses, discovering forty-one differentially expressed proteins. Furthermore, they found that the H1N1 virus led to an up-regulation of the CCR4-NOT transcription complex subunit 4, regulating the RNA degradation in mast cells in this way. Instead, the H5N1 virus down-regulates the p53-signalling pathway, suppressing apoptosis.

Tang and his group [44] recently studied the transcriptomic profile of murine mast cells infected by H1N1 and H5N1 viruses. This study identified signalling pathways that are differently expressed in the two infections. Precisely, H1N1 infection activates the foxO signalling pathway and the autophagy pathway. The foxO pathway mediates anti-apoptotic and anti-inflammatory responses, explaining why H1N1 infection is less intense than H5N1. In contrast, H5N1 infection favours the expression of NF-κB, an essential regulator of the inflammatory response and the necrosis pathway. This study, therefore, demonstrates how H5N1 activates mast cells to a greater extent than H1N1 [44].

Another key point from this study is the importance of the Nbeal2 gene. This gene is necessary for developing granules and would be involved in mast cell activation. From the analysis of the transcriptomic profile, the Nbeal2 gene is more expressed by H5N1 infected mast cells, explaining why this infection tends to be more severe than that by H1N1 [44].

Few studies have examined the role of basophils in the pathogenesis of influenza A. Clementsen and his group have extensively studied the role and activity of basophils in influenza A virus infection in the past.

Already in 1989, he discovered the virus’s ability to enhance histamine release from infected human basophils through the activity of viral neuraminidase [45].

A year later, the results were reconfirmed through a study on cells treated with anti-neuraminidase antibodies, which completely abolished the virus’s effect on histamine release [46]. The same effect was obtained using antibodies against viral hemagglutinin, demonstrating their importance in the viral mechanisms enhancing histamine release [46].

Furthermore, carbohydrates would help reduce the influenza virus’s effect on histamine release since they would block the same binding sites exploited by the virus [47].

In vitro studies by Huftel et al. [48] provided further evidence of the ability of influenza A virus to induce histamine release from antigen-stimulated human basophils. In addition, incubating human leukocytes with the virus also promotes the generation of leukotrienes (LTC4). Even more interestingly, they found that eliminating T cells from the mixture before incubation obliterated these effects, demonstrating the importance of these cells in the pathogenesis of the disease.

In a recent study, it was demonstrated that a low basophil count, associated with low levels of aspartate aminotransferase and low-density lipoprotein cholesterol, were associated with the most severe forms of influenza A H1N1pdm09 [49].

**Figure 2:**
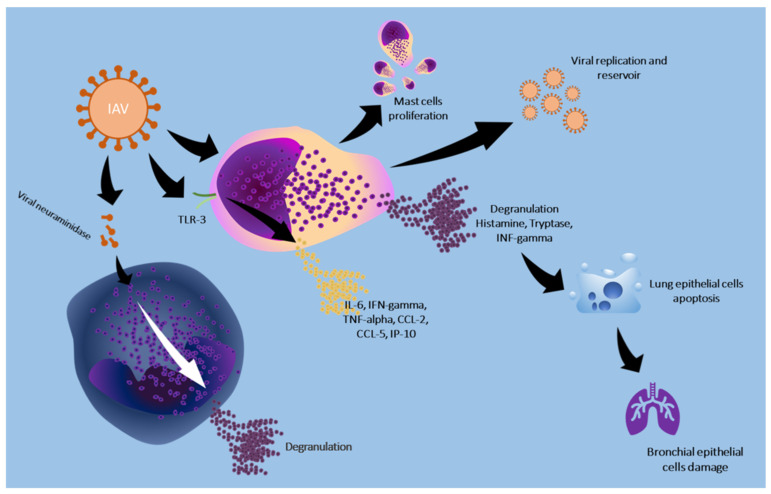
IAV causes mast cell degranulation and the release of proinflammatory cytokines through the interaction between viral proteins and TLR-3. The release of inflammatory mediators damages the respiratory epithelium and releases viruses, perpetuating the infection. Viral neuraminidases can also promote basophil degranulation, releasing other proinflammatory factors.

Figure 2 schematizes the effect of IVAs on mast cells and basophils.

Table 2 summarizes data about the role of mast cells and basophils in IAV infection.

### 3.3. Role of Mast Cells and Basophils in the Course of HIV Infection

Human immunodeficiency virus infection has long been a global public health problem, so much so that it has been considered a pandemic. It is responsible for the acquired immunodeficiency syndrome (AIDS).

Even though antiretroviral treatment reduces AIDS-related deaths, access to therapy is not equal worldwide and the prospects of curative treatments or an effective vaccine are still uncertain; as such, AIDS will continue to represent a significant public health burden [50,51].

The pathogenic mechanisms underlying infection and disease are complex and involve innate and acquired immunity.

Recent studies have shown that both basophils and mast cells capture HIV and promote trans-infection of CD4+ T cells through interaction with four proteins (gp120, gp41, Tat, and Nef) [52].

Despite numerous studies, the role of mast cells and basophils remains unclear.

A 2016 literature review [52] highlighted the potential role of these cells in the infectious process. Indeed, mast cells and basophils exposed to the virus can interact with several HIV proteins, regulating their response to infection. The key element that emerged from this review was the ability of these cells, demonstrated in numerous in vitro experiments, to transfer the virus to CD4+ T cells, thus providing a means for the virus to spread [52].

A recent review has also highlighted the role of basophils in the pathogenesis of the disease. Although considered minor players in the infectious process, these cells have been shown to have a central role since their activation creates a cascade of immune events and influences immune modulation, cytokine release, and activation of other cells of the adaptive immune system [53].

Since the 2000s, some works have demonstrated the virus’s role in inducing these cells’ degranulation. In fact, it was noted that in patients with HIV, serum IgE levels were elevated and allergic reactions were more frequent [54]. Marone et al. [55,56,57] demonstrated, through in vitro experiments, that HIV-1 gp 120 interacting with the V_H_3 region of IgE induced the release of inflammatory mediators by human mast cells and basophils. Furthermore, through in vitro studies, the same authors discovered how the Tat protein expressed by the virus stimulated the chemokine receptor CCR3 present in these cells, inducing its expression, influencing cell migration, and promoting the release of IL-4 and IL-13 [55,56,57].

In the same years, studies by Li et al. [58] also confirmed the susceptibility of human mast cells and basophils to HIV-1. In the peripheral blood of infected patients, they found metachromatic CD4+/CCR3+/CCR5+/CXCR4+ cells expressing the receptors for IgE FcεRI. During an allergic reaction, these cells would be able to release not only TNF-alpha and other inflammatory mediators but also the virus, exacerbating the infection [58].

In addition to gp41 and Tat, the Nef protein plays a role in HIV-1 pathogenesis. This protein is produced during the early stages of infection to create the optimal microenvironment for viral replication. Rossi et al. [59] studied the interaction between Nef, human basophils, and mast cells.

Their in vitro studies showed that incubating these cells with Nef stimulates the release of some chemokines (CXCL8/IL-8 and CCL3/MIP-1α), thus favouring the direct recruitment and activation of basophils and mast cells at the sites of viral replication.

The IgE-FcεRI interaction seems important, which would positively regulate the functional expression of CXCR4 on progenitor mast cells (prMC) [60]. Indeed, there are HIV variants that have a different tropism for different chemokines. CCR5-tropic variants (R5 viruses) bind CCR5, CXCR4-tropic variants (X4 viruses) bind CXCR4 and R5X4 viruses can bind both. IgE-FcεRI interactions during the mast cell ontogenesis phase increase the susceptibility of prMC to X4 and R5X4 viruses. Moreover, this effect is completely inhibited by using specific CXCR4 peptide antagonists or omalizumab (anti-IgE antibody). Thus, elevated IgE levels in HIV-positive and allergic individuals would influence the composition of viral variants stored in the tissue reservoir of long-lived mast cells [60].

Recently, Song et al. [61] identified the specific role of mast cells in acute retrovirus infection. Using a mouse model of MuLV/Friend virus infection, they demonstrated that this virus triggers mast cell degranulation, activating granulocyte-like myeloid-derived suppressor cells (G-MDSCs). These cells suppress CD8+ T-cell and NK-cell-mediated antiviral immune responses. Furthermore, the administration of mast cell stabilizers stimulates antiviral immune responses by suppressing retroviral infection [61].

One of the major chronic complications of HIV-infected patients is chronic lung disease, in which lung mast cells play a key pathogenetic role. Human lung mast cells (HLMC) express FcεRI, and it seems that the interaction with the superantigen gp120 expressed by the virus causes the release of proinflammatory mediators from HLMC. In the studies of Marone et al. [62], incubation of HLMC with gp120 also induced the release of angiogenic and lymphangiogenic factors from these cells through the interaction of gp120 with IgE VH3+ bound to FcεRI.

The role of mast cells in the pathogenesis of the disease has been better studied than that of basophils. Furthermore, a recent review of the literature has highlighted how many mast cells can be useful in the diagnosis and prognosis of the disease [63]. Among these, high levels of CD117 (c-kit) are correlated with increased proliferation and activation of mast cells in these patients, as well as high levels of tryptase reflecting disease progression or high levels of chymase (an index of fibrosis and remodelling) reflecting the state of possible complications [63].

**Figure 3:**
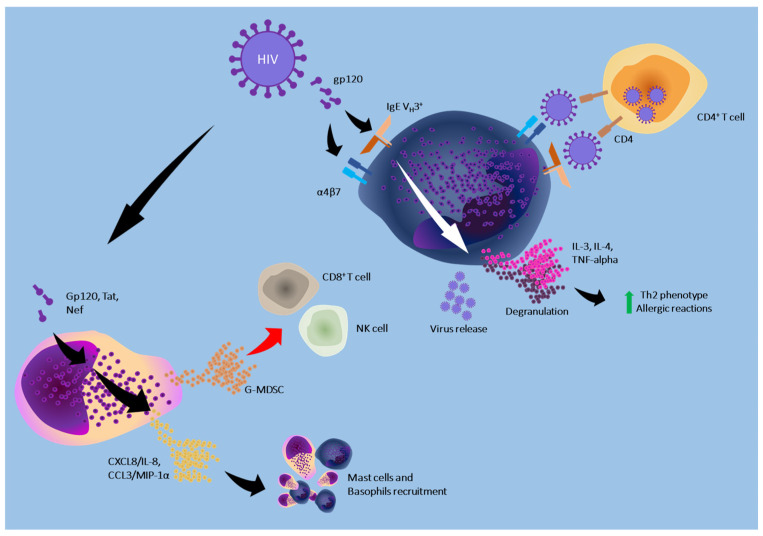
The gp120 protein expressed on the virus interacts with the V_H_3^+^ region of IgE and with α4β7 on the basophil surface, creating bridges through which the virus infects the CD4^+^ T lymphocyte. The same receptors also stimulate mast cell degranulation and release factors promoting the Th2 inflammatory phenotype. In this way, allergic phenomena are favoured. The gp120 protein, Tat, and Nef stimulate the release of chemokines by the mast cell, favouring the recruitment of mast cells and basophils and amplifying the response. In addition, the mast cell releases G-MDSC, a factor that inhibits the antiviral activity mediated by CD8^+^ T cells and NK cells; The green arrow indicates an increase, the red arrow indicates a decrease.

Figure 3 represents the mechanisms through which HIV interacts with mast cells and basophils.

Table 3 summarizes all the works regarding the role of mast cells and basophils in the infection.

### 3.4. Role of Mast Cells and Basophils in Dengue Virus Infection

The dengue virus is a single-stranded RNA virus caused by the bite of the mosquito *Aedes aegypti*, which deposits virus particles in the skin. It is distributed in tropical and subtropical areas and could potentially become a public health problem in Italy, given the increase in cases during 2024 and in consideration of the ongoing climate change [64]. Clinically, dengue can occur asymptomatically until the most severe cases, in which it causes a haemorrhagic fever with shock [65].

It would appear that mast cells are involved in the immune response during dengue infection, as high levels of histamine have been found in the blood and urine of patients with dengue haemorrhagic fever [66]. This hypothesis is supported by the fact that the initial site of infection of the dengue virus is the skin, which is, therefore, the first critical organ in the immune response to the virus and with it also the resident cells, such as skin mast cells [67]. Furthermore, endothelial cells infected with dengue virus produce chemokines that recall and activate mast cells [68].

Mast cell involvement has been related not simply to the immune response during dengue virus infection but specifically to the course and prognosis of the infection.

The role of mast cells in immune surveillance against the dengue virus would appear to unfold in several ways: through the release of preformed mediators, through the modification of gene expression by the production of chemokines that attract other immune cells, and through the recruitment of NK cells, which facilitate viral clearance [69].

A study in mice demonstrated mast cells’ crucial role in dengue’s course and prognosis. Mast cell-deficient mice inoculated with the virus exhibited increased viral replication, bleeding time, and worse prognosis than infected mice with normal mast cell numbers [70].

Mast cells fulfil their role as effectors of innate immunity in response to viral infection through the secretion of various mediators, including TNF-alpha, CCL4 and 5, and IFN-I, which all contribute to the immunoregulatory action by recruiting other cells affecting immunity, such as T lymphocytes, monocytes, and NK cells. The production of IFN-I is enough to safeguard the surrounding cells from infection [71] and to promote antiviral activity against the dengue virus [72]. Mice lacking IFN-I and II are more susceptible to dengue virus infection and have comorbidities, except for mice born from immunized mothers, despite the immunocompromise above. Moreover, it has been noted that the expression of IFN-gamma alone in the absence of IFN-I correlates with greater disease severity and increased mortality [73]. The CCL4 and five chemokines appear to have a positive prognostic significance, as their levels are reduced in patients with dengue haemorrhagic fever [74,75]. In response to dengue infection, mast cells and basophils also produce other chemokines, such as RANTES and MIP-1, that recruit effector T cells [76].

However, some studies demonstrate the role of dengue virus-infected mast cells in activating endothelial cells via increased TNF-alpha in the absence of increased permeability, whereby other mediators and interaction with other cells are required. TNF-alpha is thought to be primarily responsible for endothelial remodelling and plasma leakage in vivo during severe dengue infection [77]; chymase also contributes to dengue virus-induced vascular leakage [78] and it has been proposed as a predictive biomarker of dengue haemorrhagic fever [79,80]. Furthermore, leukotrienes and proteases released by mast cells contribute to endothelial loss. The absence of mast cells appears to be related to reduced vascular permeability during dengue infection [81]. Tryptase is a serine protease released exclusively by mast cells that contributes to the degradation of fibrinogen and coagulation factors and would appear to play a pivotal role in dengue virus-induced vascular permeability [82], especially in its alpha isoform [83]. Serotonin released by mast cells also contributes to dengue haemorrhagic fever by binding to 5HT2 receptors on platelets, which become activated, aggregate, and are sequestered by the spleen resulting in thrombocytopenia [84].

Therefore, several mediators released by mast cells and basophils, including vasoactive mediators, act in concert to determine endothelial damage in the most severe forms of dengue [85].

Mast cell activation during dengue virus infection is antibody-mediated. FcεRII receptor in its two different isoforms regulates mast cell activation and the release of mediators such as CCL5 [86]. Mast cell activation and degranulation mediated by preexisting anti-dengue antibodies could correlate with more severe forms of secondary dengue infection. Disease severity correlates with levels of phospholipase A2, whose activity is linked to that of PAF, a mediator that contributes to vascular damage in dengue haemorrhagic fever [87].

Most studies have focused on the role of mast cells in dengue virus infection, their interplay with other cells, and the correlation between mediators and cytokines released by mast cells and the course and prognosis of infection. Currently, the role of basophils in the course of dengue infection is limited and poorly understood.

**Figure 4:**
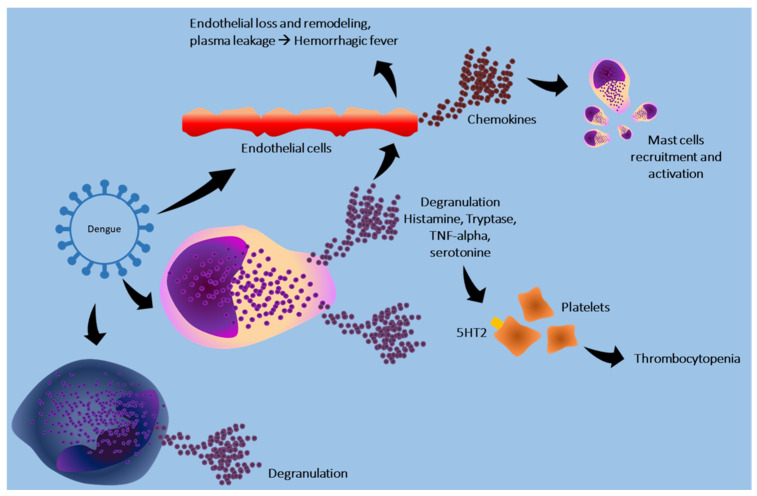
Dengue virus stimulates endothelial cells to release chemokines that attract and activate mast cells. The virus stimulates these cells and, through the release of histamine, tryptase, and TNF-alpha, promotes endothelial damage. Furthermore, the release of serotonin by mast cells determines the activation of platelets, mediated by the 5HT2 receptor, promoting splenic sequestration of platelets and thrombocytopenia. The virus also promotes basophil degranulation.

The mechanisms through which the dengue virus interacts with these cells are shown in Figure 4.

Table 4 summarizes the work on the role of mast cells and basophiles in the infection.

## 4. Discussion

Mast cells and basophils are cells of the innate immune system implicated in the adaptive Th2 response and appear to play a role in many viral infections [88,89]. This research has shed new light on the involvement of these cells in SARS-CoV-2 infection.

Mast cells are active in COVID-19 infection, particularly in determining lung damage, by producing proinflammatory cytokines [1]. New data have supported these results after the review mentioned above [13]. In particular, it has been seen how the spike glycoprotein of SARS-CoV-2 can induce mast cell degranulation.

Other authors have shown how changes in gene expression during infection can be useful to evaluate the prognosis of patients with SARS-CoV-2 infection.

As we previously reported [1], basophils are important both in the acute phase of the disease and in remission. In the acute phase, particularly in the most aggressive forms, a reduction in the number of basophils and the expression of CRTH2 is observed, with a significant increase in PD-L1. In advanced stages of the disease, basophils increase in number and help the humoral and cellular immune response by producing IL-4. More recent studies have also demonstrated the importance of basophils stimulated by IL-3 during infection. These cells produce large amounts of IL-13, which would be implicated in reducing inflammation and stimulating antibody production [19].

Many retrospective studies have focused on the possibility of using different laboratory indices as markers of disease or disease severity or even as indicators of risk of serious disease. However, the results regarding the role of basophils as indicators are conflicting in the literature and therefore further studies are required for them to be used for this purpose [21,22,23,24,25].

On the contrary, the use of machine learning for the creation of diagnostic and prognostic algorithms of disease from which a relevant role of basophils emerges is interesting [27,28,29,30,31].

This review shows how mast cells’ role also seems relevant to the immune response to influenza viruses. Several authors have recently studied the topic and there is a strong correlation between influenza A viruses and mast cells. Mast cells are mainly involved in lung damage that presents itself in the most severe forms of infection. 

The virus promotes the release of inflammatory mediators, such as histamine, tryptase, and IFN-gamma, the latter being responsible for the apoptosis of respiratory epithelial cells and lung damage in the most severe forms of the disease. The identified pathways involved are RIG-I/MAVS and TRL-3.

In vivo studies on experimental animals of disease that have been effectively pharmacologically treated with molecules that act on the mastocyte support these data [40,41].

Several authors have evaluated the gene expression of these cells during infection compared to the cells of healthy subjects, discovering how the gene expression was profoundly different between infected and healthy cells and between mast cells infected with other subtypes of IAV.

While studies on mast cells are numerous, recent studies on the role of basophils in the pathogenesis of IAV are few.

These mainly concern the role of histamine and other inflammation mediators released by the basophils stimulated by the virus through the activity of neuroaminidase and haemagglutinin [46]. Also interesting is the protective role of the carbohydrates that would saturate the same receptors stimulated by the virus [47].

The possibility of using basophils as a prognostic indicator of illness has recently been assessed. Indeed, low basophil counts could be associated with the most severe forms of the disease [49].

Although the global impact of HIV is relevant, the scientific interest in the role of cells of innate immunity in the pathogenesis of the disease seems to have reduced in recent years.

In fact, until 2016, the interest of the scientific community towards mast cells and basophils was significantly greater than in recent years. Until then the study of the role of these cells in the infection were mainly aimed at their ability to transfer the virus to the CD4+ T cells [52]. Indeed, mast cells and basophils can release the virus in the circle and represents an endogenous source [58].

Several authors have also shown that HIV can induce the liberation of inflammation mediators through interaction with IgE linked to the membrane receptors present on basophils and mast cells. These results explain why these subjects are more sensitive to the development of allergic reactions to drugs.

Human lung mast cells, responsible for lung damage in immunocompromised patients, express FCεRI and is the interaction between IgE of VH3-family and the gp120 superantigene expressed by the virus that causes the release of proinflammatory mediators, angiogenetic and lymphangiogenetic factors by these cells, exacerbating the damage [62].

Mast cells play an active role in immune surveillance against the dengue virus, through the release of proinflammatory mediators, chemokine and the recruitment of NK cells.

Mast cells exercise their action through the secretion of TNF-alpha, CCL4 and 5, IFN and the recruitment of T lymphocytes, monocytes and NK cells. In response to the infection, mast cells together with basophils also produce other chemokines such as Vipers and Mip-1 with the ability to encourage the recruitment of the effector T cells [76]. Several mediators issued by these cells act together to determine the endothelial damage in the most serious forms of dengue [85].

## 5. Conclusions

Knowing the mechanisms behind the high contagiousness of viral infectious pathologies is essential to finding new ways to treat them. The role of innate immunity cells is often underestimated, but this knowledge could be valuable in facilitating the diagnostic–therapeutic process. Our analysis showed that mast cells and basophils play crucial roles in developing viral diseases, as we described. The mediators released by the degranulation of these cells following viral stimulation are implicated in inflammatory processes and could be a potential therapeutic target for preventing severe forms or complications related to the disease. The count of these cells associated with other clinical or laboratory indices could be useful for obtaining prognostic scores and the prognostic value could be more precise with input from ML algorithms. While mast cell studies are varied, those on basophils are limited, especially in studying HIV, IAVs, and the dengue virus, and further insights are needed. In the future, understanding these mechanisms better could lead to the development of innovative therapies and sensitive diagnostic tools that could help reduce the mortality linked to these pathologies.

## Figures and Tables

**Table 1:** Mast cells and basophils in SARS-CoV-2 infection.

Author, Year	Cell	Type of Study	Test Subject	Results
Li et al., 2023 [12]	Mast cell	Transcriptomics analysis	Human blood samples	NCAPG, MCM10 and CDC25C were identified as hub genes in peripheral blood, which could be used as diagnostic markers of poor prognosis
Tan et al., 2023 [13]	Mast cell	In vitro/transcriptomics analysis	Mice, non-human primate/human blood samples	Mouse chymase (MCPT1) levels were significantly elevated days 1, 3, 5, and 7 after SARS-CoV-2 infection. Infected primates experience lung pathology involving haemorrhagic manifestations and widespread MC activation. Patients with COVID-19 have significantly higher chymase serum level compared with controls (the highest levels were detected in severe group)
Gebremeskel et al., 2021 [14]	Mast cell	In vitro	Human blood samples	Patients with SARS-CoV-2 have significantly higher levels of CCL2, CCL3, CCL4, IP-10, IL-6, IL-8, VEGF, TNF-alpha, IFN-gamma, chymase, β-tryptase, and CPA3, suggesting systemic mast cell activation. Mast cell-derived proteases and eosinophil-associated mediators are increased in the lungs and/or serum of patients with COVID-19
Cao et al., 2024 [15]	Mast cell	In vitro	Mice	SARS-CoV-2 infection induces mast cell accumulation and degranulation in the peri-trachea in mice. Mast cell activation induces the production of inflammatory factors in bronchial epithelial cells. Ebastine or loratadine reduces the induction of inflammatory factors and alleviate tracheal injury in mice
MacCann et al., 2023 [16]	Mast cell	In vitro	Human blood samples	Moderate/severe COVID-19 group, showed greater downregulation of FCER1A (gene encodes for FcεRI on mast cells) than the group with mild disease. Downregulation of the TNF-alpha, PTGS2 and IL1B genes was observed in the moderate/severe COVID-19 group, suggesting downregulation of mast cells activation. Circulating levels of IL-1β, IL-6, IL-17A and IL-10 were significantly elevated in the COVID-19 group compared to the SARS- group
Wu et al., 2021 [17]	Mast cell	In vitro	Mouse and non-human primate mast cells	Lung lesions, including inflammatory cells (lymphocytes and monocytes) infiltration, haemorrhage, alveolar septal thickening, and mucosa desquamation were observed around the areas of mast cell accumulation and degranulation in both mice and macaques, compared with controls. Spike-RBD binding to ACE2 is accompanied by an immediate mast cells degranulation, by the release of intracellular tryptase and chymase
Liu et al., 2022 [18]	Mast cell	In vivo (animal model)	Mice	SARS-CoV-2 spike glycoprotein triggers mast cell activation. MCP2 chymase has formed a complex with the spike protein, promoting protease-dependent viral entry. Mast cell stabilizers or chymase inhibitors reduce viral entry. The absence of mast cells affects early viral load in the upper respiratory tract, increasing the risk of viral invasion into the lower respiratory system
Reddy Bonam et al., 2022 [19]	Basophil	In vitro	Human blood samples	SARS-CoV-2 induces the release of IL-13 from basophils. The virus or infected epithelial cells do not alter the expression of CD69, CD13, or CD107a. SARS-CoV-2 does not induce PD-L1 on basophils
Degenfeld-Schonburg et al., 2024 [20]	Mast cell and Basophil	In vitro	Human blood samples	Primary mast cells, basophils, and eosinophils and their corresponding cell lines express CD13 and CD147 of CoV-R. Primary skin mast cells and basophils, as well as EOL-1 cells also express CD26, whereas the cell lines do not
Liu et al., 2023 [21]	Basophil	In vitro	Human blood samples	Increased levels of basophils, associated with other parameters including advanced age, high fever, increased white blood cells, CRP, LDH, high-sensitivity troponin, pro-BNP, and D-dimer are associated with severe disease and worse prognosis
Lin et al., 2024 [22]	Basophil	In vitro	Human blood samples	Higher levels of basophils (%) and monocytes (%) are associated with an increased risk of severe infection
Binsaleh et al., 2023 [23]	Basophil	Data analysis	Human blood samples	Disease-related mortality was significantly associated with a reduction in white blood cells and basophils
Ben et al., [24] 2024	Basophil	Data analysis	Human blood samples	Individuals with non-severe disease showed reduced levels of basophils and basophils (%). Basophil counts combined with lymphocytes or platelet-to-lymphocyte ratio were more sensitive for detecting non-severe cases early
Rauf Saeed and Lal 2023 [25]	Basophil	Data analysis	Human blood samples	No correlations were found between basophil values and disease severity
Fu et al., 2022 [27]	Basophil	Data analysis	Human blood samples	The predictive model based on LASSO logistic regression identified 28 variables useful for the prognosis of critical illness. Among these factors, a low basophil count is among the parameters associated with the development of critical illness at the time of hospital admission
Chadaga et al., 2024 [28]	Basophil	Data analysis	Human blood samples	Basophils together with CRP, lymphocytes, albumin, D-dimer, and neutrophils have proven to be the best predictive markers of severity
De Souza et al., 2023 [29]	Basophil	Data analysis	Human blood samples	The unsupervised clustering analysis has been shown to be effective in facilitating the decision-making process in the patient with suspected infection through the use of simple parameters such as leukocytes, basophils, eosinophils, and red cell distribution width
Wang et al., 2022 [30]	Basophil	Data analysis	Human blood samples	Basophil levels associated with mean corpuscular volume, red blood cell distribution width, and platelet distribution width may be useful in predicting recovery in patients with moderate COVID-19. Small increases in these parameters within normal limits suggest improvement in these patients
Min Baik et al., 2023 [31]	Basophil	Data analysis	Human blood samples	Basophils, D-dimer, eosinophils, glucose, and aspartate aminotransferase are excellent markers for differentiating the two conditions

**Table 2:** Mast cells and basophils in IAV infection.

Authors, Year	Cell	Type of Study	Test Subject	Results
Ng et al., 2019 [34]	Mast cell	In vitro	Human mast cell (LAD2), human lung epithelial cell (Calu-3)	The three strains studied did not induce the release of histamine or β-hexosaminidase in LAD2. A/HK/8/68 induced the release of prostaglandin D2 in LAD2. CCL4 was released in TLR in a statistically significant way from LAD2 cells infected with A/PR/8/34. Increased expression of viral recognition receptors (RIG-I and MDA5) and viperin mRNA was found
Hu et al., 2012 [35]	Mast cell	In vitro/In vivo (animal model)	Mice/P815 cell line	Mast cells are significantly activated by the virus which promotes the release of histamine, tryptase and IFN-gamma
Graham et al., 2013 [36]	Mast cell	Ex vivo	C57BL/6 mice, B6.Cg-Kit(W-sh) mice and derived mast cells	A/WSN/33 causes the release of cytokines and chemokines from mast cells through a RIG-I/MAVS-dependent mechanism. Histamine release is independent of this mechanism
Meng et al., 2017 [37]	Mast cell	In vitro/In vivo (animal model)	P815 cell line, mice	H1N1, H5N1 and H7N2 viruses activated infected P815 cells via the Toll-like receptor 3 pathway, stimulating the production and release of proinflammatory cytokines and chemokines (IL-6, IFN-gamma, TNF-alpha, CCL-2, CCL-5 and IP-10)
Liu et al., 2014 [38]	Mast cell	In vitro	P815 cell line	Influenza viruses H1N1 (A/WSN/33), H5N1 (A/Chicken/Henan/1/04), and H7N2 (A/Chicken/Hebei/2/02) induce mast cell apoptosis through the intrinsic mitochondria/cytochrome c pathway
Desheva et al., 2020 [39]	Mast cell	In vivo/Ex vivo	Mice	Sixty-seven percent of vaccinated mice were protected from lethality compared to forty-three percent in the placebo group. Administration of antihistamines increased survival to 85–95%. More active mast cells were found in the lungs of immunized mice.
Han et al., 2016 [40]	Mast cell	In vivo (animal model)	Mice	The survival rate in treated mice was higher than in untreated mice and the expression of IL-6, TNF-alpha, TLR-3, and TIR-domain-containing adapter-inducing interferon-β was significantly reduced
Huo et al., 2023 [41]	Mast cell	In vivo (animal model)	AANAT−/− melatonin-deficient mice, wild type mice	Melatonin suppresses alveolar epithelial cell apoptosis both in vitro and in vitro, decreasing lung damage during infection. Melatonin suppresses the HIF-1 pathway and inhibits the release of proinflammatory cytokines from mast cells
Huo et al., 2019 [42]	Mast cell	In vitro	P815 cell line	The 5-HT and cyclic guanosine monophosphate (cGMP)/protein kinase G (PKG) signalling pathways are activated primarily in H1N1-infected P815 cells. The HIF-1 signalling pathway is preferentially activated in H7N2-infected P815 cells. Corresponding mRNA levels are also increased.
Wu et al., 2019 [43]	Mast cell	In vitro	Human mast cells	Forty-one differentially expressed proteins in human mast cells have been related to infection by H5N1 versus seasonal H1N1 virus. H1N1 significantly regulates the RNA degradation pathway via positive regulation of the CCR4-NOT transcription complex subunit 4. H5N1 suppresses apoptosis via negative regulation of the tumour protein p53 signalling pathway. The hypoxia-inducible factor-1 signalling pathway is more susceptible to H5N1 infection than to H1N1 virus
Tang et al., 2022 [44]	Mast cell	In vitro	P815 cell line	H1N1-infected mouse P815 mast cells exhibit more upregulated and downregulated genes than H5N1-infected cells. Differentially expressed genes in H1N1 infection were specifically enriched for FoxO and autophagy pathways. Differentially expressed genes in H5N1 infection were specifically enriched for NF-κB and necroptosis pathways. Nbeal2 is preferentially activated in H5N1-infected P815 cells
Clementsen et al., 1988 [45]	Basophil	In vivo (human)	Human basophils	Viral neuraminidase enhances histamine release from basophils
Clementsen et al., 1989 [46]	Basophil	In vivo (human)	Human basophils	In order for neuroaminidase to promote basophil activation, the virus must bind to some cell surface protein
Clementsen et al., 1990 [47]	Basophil	In vivo (human)	Human basophils	Influenza A virus causes an enhancement of mediator release. The enhancement is abolished by galactose, N-acetylglucosamine, alpha-methyl-D-glucoside, alpha-methyl-D-mannoside, N-acetylneuraminic acid and lactose. Sugars prevent the enhancement of mediator release by binding to the cell membrane of basophils
Huftel et al., 1992 [48]	Basophil	In vitro	Human mononuclear cells	Incubation with influenza A stimulates the release of LTC4 and histamine from basophils. Histamine release was enhanced in the virus-treated mononuclear cell group that had not undergone T-cell depletion
Wang et al., 2024 [49]	Basophil	In vitro	Human blood samples	Inverse variance weighted analysis revealed a correlation between low AST, LDL-C, and basophil levels with severe H1N1pdm09 disease.

**Table 3:** Mast cells and basophils in HIV infection.

Authors, Year	Cell	Type of Study	Test Subject	Results
Marone et al., 2001 [55]	Mast cell and Basophil	In vitro	Human mast cells and basophils	HIV-1 proteins gp120 and Tat trigger the release of proinflammatory cytokines and polarize the T(H)2 cell response through FcεRI (+) cells and the beta-chemokine receptor CCR3 on these cells
Li et al., 2001 [58]	Mast cell Basophil	Ex vivo/In vitro	Human mast cells and basophils	Metachromatic cells express on their surface FcεRI, CD4, and the chemokine receptors CCR3, CCR5, and CXCR4, but not CD3 and CD68. These cells are susceptible to HIV-1
Rossi et al., 2016 [59]	Mast cell Basophil	In vitro	Human mast cells and basophils	Incubation of basophils and mast cells with Nef induced the release of CXCL8/IL-8 and CCL3/MIP-1α. Nef protein has a crucial role in basophils and mast cells recruitment at site of virus replication
Sundstrom et al., 2009 [60]	Mast cell	In vitro	Human progenitor mast cells	The interaction between IgE and their receptor, in the phases of mast cell ontogenesis, would positively regulate the functional expression of chemokines, influencing the composition of viral variants stored in the tissue reservoir of long-lived mast cells
Song et al., 2022 [61]	Mast cell	In vivo (animal model)	C57BL/6 wild type mice, C57BL/6-*Kit ^W^^-^^sh/W^*^-^*^sh^* (Sash) mice	In in vivo models, retrovirus infection stimulates mast cell degranulation through activation of G-MDSCs
Marone et al., 2020 [62]	Mast cell	In vitro	Human lung mast cells	gp120 acts as a superantigen, interacting with FcεRI-bound IgE of V_H_3 family of mast cells and promoting the release of proinflammatory mediators, angiogenic and lymphangiogenic factors

**Table 4:** Role of mast cells and basophils in dengue infection.

Authors, Year	Cell	Type of Study	Test Subject	Results
Troupin et al., 2016 [67]	Mast cell	In vitro	Human skin mast cells	The skin and its resident cells, such as mast cells, are the first critical organ in the immune response to the dengue virus
Dalrymple et al., 2012 [68]	Mast cell	In vitro	Human endothelial cells	Endothelial cells infected with dengue virus produce chemokines that recall and activate mast cells
St. John et al., 2011 [69]	Mast cell	In vitro	Human, mice and monkey mast cells	Immune surveillance through the release of preformed mediators, the modification of gene expression by chemokines production and the recruitment of NK cells
Chu et al., 2015 [70]	Mast cell	In vivo (animal model)	Kit (W-sh/W-sh) mice	Mast cell-deficient mouse models show increased infection and macrophage infiltration at the skin injection site, as well as bleeding time compared to wild-type mice.
Brown et al., 2012 [71]	Mast cell	In vitro	Human cord blood-derived mast cells, KU812 and HMC-1 mast cell lines	Immunomodulation of antiviral response via secretion of IFN-I, TNF-alpha, and CCL4 and 5
Morrison et al., 2017 [72]	Mast cell	In vivo (animal model)	Mice	Mast cell membrane stabilizers enhance the antiviral activity of the immune system through the release of INF-I
Mantri et al., 2021 [73]	Mast cell	In vivo (animal model)	Mast cell-deficient mouse model	IFN-I and II deficiency is related to comorbidity; IFN-gamma expression without IFN-I expression is related to increased mortality
Bozza et al., 2008 [74]	Mast cell	In vitro	Human blood samples	CCL4 and 5 chemokines are reduced in patients with dengue haemorrhagic fever
King et al., 2002 [76]	Mast cell and Basophil	In vitro	Human mast cells and basophils	Elevated levels of RANTES, MIP-1alpha, and MIP-1beta were observed following infection. Levels of IL-8 and ENA-78 were not increased
Brown et al., 2011 [77]	Mast cell	In vitro	Human cord blood-derived mast cells, HMC-1	Infection results in the release of factors that activate human endothelial cells and increased expression of the adhesion molecules ICAM-1 and VCAM-1. Use of a specific TNF-blocking antibody blocked this effect, identifying TNF as an endothelial cell activating factor
Chu et al., 2017 [78]	Mast cell	In vivo (animal model)	Mice	Chymase contributes to dengue virus-induced vascular leakage
St. John et al., 2013 [81]	Mast cell	In vivo (animal model)	Mice	Leukotriens and proteases released by mast cells contribute to vascular damage
Rathore et al., 2019 [82]	Mast cell	In vitro	Human mast cells	Tryptase contributes to the degradation of fibrinogen and coagulation factors and probably to dengue virus-induced vascular permeability.
Masri et al., 2019 [84]	Mast cell	In vivo (animal model)	Mast cell-deficient mice, wild-type mice	Serotonin released by mast cells contributes to thrombocytopenia in dengue haemorrhagic fever
King et al., 2000 [85]	Mast cell and Basophil	In vitro	Human Mast cells and basophils	Vasoactive cytokine production by mast cells/basophils may contribute to the vascular pathology seen in severe dengue disease
Brown et al., 2006 [86]	Mast cell	In vitro	Human Mast cells	Mast cell activation is antibody-mediated via FcεRII
Jeewandara et al., 2016 [87]	Mast cell	In vivo (human)	Human blood samples	Phospholipase A2 activity significantly correlates with the degree of viraemia in patients with dengue haemorrhagic fever

## Data Availability

Not applicable.
